# Parenting a Toddler in the Era of Pervasive Screens: Interviews with Low-Income Mexican American Parents

**DOI:** 10.3390/ijerph20085461

**Published:** 2023-04-11

**Authors:** Darcy A. Thompson, Andrea M. Jimenez-Zambrano, Haley Ringwood, Jeanne M. Tschann, Lauren Clark

**Affiliations:** 1 Department of Pediatrics, University of Colorado Anschutz Medical Campus, 12631 East 17th Ave., Aurora, CO 80045, USA; andrea.jimenez-zambrano@cuanschutz.edu; 2 Adult and Child Center for Health Outcomes Research and Delivery Science, University of Colorado Anschutz Medical Campus, 1890 N Revere Ct, Aurora, CO 80045, USA; 3 Denver Health and Hospital Authority, 777 Bannock Street, Denver, CO 80204, USA; haley.ringwood@dhha.org; 4 Department of Family Medicine, University of Colorado Anschutz Medical Campus, 12631 East 17th Ave., Aurora, CO 80045, USA; 5 Department of Psychiatry and Behavioral Sciences, University of California at San Francisco, San Francisco, CA 94143, USA; jeanne.tschann@ucsf.edu; 6 School of Nursing, University of California, 700 Tiverton Avenue, Factor Building, Los Angeles, CA 90095, USA; lclark@sonnet.ucla.edu

**Keywords:** parenting, screen time, digital media, health disparities, qualitative, Latino, Hispanic, behavior management, beliefs, monitoring

## Abstract

Screen media use starts in early childhood, despite recommendations to limit use. This study explored low-income Mexican American mothers’ and fathers’ beliefs, parenting practices, and perceived contextual contributors related to toddler screen use. We conducted interviews with 32 low-income Mexican American parents. Transcripts of audio recordings were analyzed to identify themes. Parents perceived numerous benefits to screen use, including learning and enjoyment, as well as seeing it as a helpful tool for parents. Reported risks included harmful mental and physical effects and a risk of use being all-consuming. Parents managed screen use with a variety of practices, including giving close attention to content, monitoring duration, and engaging in co-use. They also used screens for behavior management and in specific situations, such as to prepare for sleep. Some differences in beliefs and parenting practices exist by screen device type. Parents also reported that contextual factors, such as weather and neighborhood safety, influenced screen use. This study extends the current literature regarding child screen use, with its focus on low-income Mexican American toddlers. The findings offer interventionists and providers insight into the management of screens in the lives of this population.

## 1. Introduction

Screen media use in the United States starts in early childhood, despite recommendations from national and international organizations to limit screen use (e.g., limit use to less than 1 h a day in children aged 2–5 years old) [1,2,3,4]. Today, over 80% of children <3 years old use screen devices (e.g., television (TV), tablet) [5,6]. During these early years, screen use warrants particular attention, given that screens can impede a toddler’s social interactions and physical exploration of the world, both of which support typical development during early childhood [2].

Evidence suggests that Latinx/Hispanic children spend more time using screen devices daily compared to non-Hispanic White children [7]. (For information on the terms Latinx and Hispanic, see the note at end of manuscript). The same holds true for children in low-income versus higher-income households. In fact, average daily screen use by children in low-income households is increasing, with recent evidence suggesting an almost 2-h difference in screen use duration in young children from low-income versus higher-income homes (3 h and 48 min versus 1 h and 52 min) [7]. Based on these disparities, attention to screen use in toddlers from low-income Latino families is needed.

Parents are the most proximal influence on young children and play an important role in shaping screen use, thus an understanding of parental screen-related beliefs and parenting practices in low-income Latino parents is important to inform interventions aiming to promote healthy screen use in this community. To date, however, only a handful of studies have reported on parental screen-related beliefs and parenting practices in Latino/Hispanic parents of children less than 5 years old. Beck et al. explored these topics in low-income Latino parents (mainly mothers) of 6–36 month olds, finding that parents perceived numerous benefits (e.g., educational) and potential harms (e.g., poor vision, diminishes child’s desire to engage in other activities) of TV viewing in early childhood [8]. In our own work in low-income Mexican American mothers of preschoolers aged 3 to 5 years, parents had strong feelings about the positive (e.g., educational, entertaining) and negative (e.g., imitation of bad behavior, poor vision) impacts of the content their child viewed on TV. TV viewing was also seen as helpful to mothers trying to accomplish their household tasks. Parents commonly limited the type of content their child viewed on TV, but time restrictions were not clearly defined [9]. Ochoa et. al. focused on mobile screen use in children under five in Latine families of diverse socioeconomic statuses (SES), reporting that parents (both mothers and fathers) perceived positive learning benefits of mobile screen use, but were concerned about such devices limiting social interactions and causing possible dependence. Parents reported setting time limits and monitoring use [10]. Further investigation is needed to explore parental beliefs and parenting practices in low-income Latino parents of toddlers (1–2 years old). Moreover, since most research on screen use has focused on only one screen device type or on screens as a general category, exploring possible differences in parental beliefs and practices by screen device type (TV vs. smartphones) could contribute to our understanding of toddler screen use and inform intervention design. Likewise, to ensure a comprehensive understanding of this topic, fathers should be included, especially since the majority of Latino/Hispanic children are born into two-parent households [11,12]. Currently, very little is known about how fathers shape child screen use, despite early work suggesting they have a role (e.g., paternal viewing time is associated with child viewing time).

As parenting does not occur in a vacuum [13], an understanding of contextual factors potentially influencing the parental management of toddler screen use in this population is necessary. Beck et al. reported that parents identified the type of housing as a barrier to limiting screen use [8]. Other care providers are also perceived as influencing a young child’s screen use, including extended family members [8,14]. Weather and availability of technology are also reported to influence young children’s screen use [14]. There are clearly a variety of perceived contextual contributors, thus underscoring the need to explore the influence of context on screen use in low-income Mexican American families with toddlers.

The social ecological model posits that individual behaviors and outcomes are influenced by multiple concentric levels of influence beyond individual characteristics [15,16]. Most proximally, the family influences child health through parental cultural beliefs and practices and familial patterns of health behavior. This family ‘microsystem’ is influenced by broader structures at both the community and society levels [17,18,19]. Applying the social ecological model to toddler screen use highlights the need to examine screen use within the family and the larger context of the child. More specifically, the model along with existing evidence supports the need to further understand parental screen-related beliefs, parenting practices, and contextual contributors to screen use in low-income Latino families with toddlers.

The aim of this study was to understand parental screen-related beliefs and parenting practices as well as contextual influences in low-income Mexican American mothers and fathers of toddlers. We used qualitative interviews since this approach allows an exploration of this topic in a group currently not well-represented in the literature. This study focused specifically on Mexican American parents because health beliefs and behaviors among Latino/Hispanic individuals vary and have different relationships by country of origin. Mexican Americans are the largest group of Latinos by country of origin, representing 65% of Latinos in the United States [20,21]. Guided by current evidence and the social ecological model, we addressed three research questions in this study. First, what are parents’ beliefs regarding screen use for their toddlers and do these beliefs differ by screen device type? Second, what screen-related parenting practices do parents of toddlers use and do these differ by screen device type? As parents are the most proximal influence on young children, understanding their beliefs and parenting practices regarding screen use is important to informing intervention design. The third question we addressed is what are the perceived contextual contributors to toddler screen use in parents of very young children? Findings of this study will inform cultural- and contextual tailoring of future interventions aiming to promote healthy screen use in this population.

## 2. Materials and Methods

### 2.1. Design

Informed by the tenets of focused ethnography [22,23,24], and attentive to the historical cultural, linguistic, and social organization of the Mexican American community in this location, this study used a time-intense period data collection featuring parent interviews in the home environment. Interviews were in-depth, semi-structured, and designed to explore low-income Mexican American mothers’ and fathers’ perspectives on their own and their toddler’s screen use. The study was reviewed by the Colorado Multiple Institutional Review Board and certified as exempt research. This study is part of a larger research project focused on understanding screen use in low-income Mexican American families with toddlers [25].

### 2.2. Setting and Participants

The study was conducted in the greater Denver metropolitan area, with participants recruited from the waiting room of a general pediatrics clinic located in a federally qualified health center where nearly all families served have incomes at or below the federal poverty level. The study focused on low-income families due to higher screen use in low-income vs. higher-income households [7]. We recruited adult primary caregivers (e.g., mothers, fathers, grandparents) with a child 15–26 months old who had a screen device in the home and an absence of health conditions interfering with screen use. An inclusion criterion for individuals in maternal roles was self-reported Mexican heritage. Because it can be difficult to enroll fathers using in-clinic recruitment, staff focused on recruiting partners of participating women. Due to this, fathers did not need to identify as being of Mexican descent in order to be eligible, but rather were eligible if they were partnered with a woman of Mexican descent. Of enrolled fathers, all but one identified as being of Mexican descent. Purposeful sampling, used in this study, recruits participants from theoretically important dimensions of variation [26]. Those dimensions were caregiving role (mother or father role) and language (English or Spanish). Participants were enrolled into three groups: Mothers interviewed in English, Mothers interviewed in Spanish, and Fathers. Sampling by caregiving role was employed to ensure representation of both parents. Sampling by language was performed to ensure findings represent the broader low-income Mexican American community in which language preferences vary. This intentional sampling allowed the opportunity to look for variation across these groups. Due to challenges enrolling fathers, they were not grouped separately by language. Sample adequacy and conceptual saturation were achieved after 32 participants were interviewed [27].

### 2.3. Procedures

The investigative team developed the semi-structured interview guide, informed by the social ecological model, which highlights the multiple levels of influence on behavior [15]. Adjustments were made to the interview guide following the pilot testing with three mothers who met the inclusion criteria. The guide focused on parents’ beliefs about screen use, parenting practices related to their child’s screen use, and family and neighborhood contributors to screen use. Participants were asked to identify the two top screen-device types used by their child. Following this, interviewers asked questions to elicit beliefs and parenting practices for each device. For example, they were asked what they liked and disliked about each screen device and how they managed their child’s screen use.

Following informed consent, two trained bilingual, bicultural Latina research assistants (RAs) conducted an in-person interview with each parent separately for approximately one hour. A qualitative methodologist trained the interviewers using best practices, including direct instruction, role plays, coaching, pilot interviews with feedback, and periodic debriefings post-interview [28]. Each interview started with the oral administration of a brief demographic survey, followed by a conversation guided by the semi-structured interview guide [28]. Interviews were conducted between March 2019 and April 2020 in the home setting, with one exception. The last parent interviewed in the study participated by phone due to a pandemic-related stay-at-home order.

### 2.4. Data Analysis

Interviews were audio recorded, professionally transcribed in the language of the interview (English or Spanish), and then anonymized. Spanish-language interview transcripts were then professionally translated into English. Transcripts were verified for accuracy against audio recordings and minor corrections made by the interviewer as needed. To support analysis, Spanish and English transcripts were presented side-by-side for each interview conducted in Spanish [29].

We developed a codebook using qualitative team-based research processes to engage all team members in the entire process of analysis [30]. Based on our prior work on preschooler screen use [9,31,32], we created a start-list of codes regarding screen-related beliefs and parenting practices. We included codes to identify the type of screen device being discussed (e.g., TV, smartphone). Codes were also developed inductively and defined in Atlas.ti (Atlas.ti v8 software) for consistency of application. Analysis proceeded with the refinement of codes, code definitions, and memos as researchers applied codes to transcripts. Next, focused codes and theoretical codes were applied to aid in team-based thematic analysis within and across each participant group (mothers interviewed in English, mothers interviewed in Spanish, and fathers) [33]. Throughout this process, two RAs independently coded each transcript. At monthly intervals, discrepancies in code application were reviewed in a team meeting and resolved through refinement of coding processes and discussion of differences to achieve >90% code congruence. Coded transcript data for each participant group were aggregated and then analyzed for similarities and differences by group, with particular attention to codes within the domains of parenting beliefs, parenting practices, and contextual influences. At this stage, team analysis discussions deepened interpretation of the meaning of coded data by domain. Within these domains, additional attention was given to screen device type to evaluate whether any differences existed by screen device type.

## 3. Results

Thirty-two individuals were interviewed, including eleven mothers interviewed in English, eleven mothers interviewed in Spanish, seven fathers interviewed in English, and three fathers interviewed in Spanish. Of note, one participant in the maternal role was the grandmother of the focal child. Participant and household characteristics are outlined in Table 1. Participants were on average 29 years old (SD = 6.4). Twenty-four participants (75%) reported their highest educational level as high school or less. All focal children used screen devices; average daily hours and ranges of focal child screen-use by weekend and weekday are shown in Table 1. On average, participants reported that focal children viewed more than one hour daily on both weekdays (average 2.1 h, SD = 1.4) and weekends (average 1.7 h, SD = 1.4). All participants identified smartphones as one of the top two devices used by their child, with the other device in the top two being TV, except in five households that identified tablets as the other ‘top two’ device.

### 3.1. Research Question 1: What Are Parents’ Beliefs regarding Screen Use for Their Toddlers and Do These Beliefs Differ by Screen Device Type?

Two main categories of parental beliefs were identified: The benefits of screen use and the risks of screen use. Themes within these categories are outlined below. Representative quotes for each benefit and risk, including perceived device-specific benefits, are in Table 2.

Of note, all belief findings were represented across participant groups. Through analysis evaluating for similarities and differences across participant groups, we found no systematic differences in screen-related beliefs by language (Spanish, English) or parental role (mother, father).

**Benefits of use.** Parents reported numerous benefits related to the content their child sees and how their toddler uses screen devices.
  ***Learning.*** Parents reported that toddlers learn from the content they view or interact with on screen devices, perceiving that this positively promoted their toddler’s overall development (e.g., learning sounds or words in English and Spanish; learning colors, shapes, and specific content such as animal names). Parents thought that toddlers could learn prosocial behaviors from screen use, and that physical development and activity are promoted through specific content encouraging interaction and toddler imitation (e.g., dancing).
    *Device-specific learning benefits*: Parents reported two secondary benefits of using a smartphone including children’s ability to manipulate such devices and, for parents, the benefit of children’s independent use of smartphones.
  ***Enjoyment.*** Parents shared that their children enjoy the use of screens, have fun when using them, and as a result are happy. Parents reported that co-using a screen device, involving either the parent with the child or the family all together, can be enjoyable. Parents considered co-use as a time of togetherness for the parent and child, a time that allows for cuddling (with a few comments from mothers on how this was the child’s together time with father), or as a time of positive parent–child interaction regarding shared content viewing.
    *Device-specific enjoyment benefits*: The positive sense of togetherness that parents reported was usually related to TV viewing with their child. However, some parents expressed connecting with their child simply by hearing the audio from content the child views on a different type of screen device.
  ***Helpfulness.*** Parents viewed screens as helpful tools for occupying their child’s attention and for behavior management. Screens are viewed as useful for babysitter-like functions, helping to keep a child entertained and/or preventing challenging behavior when at home or away from home (e.g., car, grocery, errands) and when parents need to do other things (e.g., chores, self-care). According to participants, screen use can be relaxing, helping a child to sit still or stay calm for a period of time when needed by the parent. Screens can also be calming before sleep or for an upset child. Screens can also keep a child ‘safe’ when a parent is engaged in certain activities, such as cooking at a hot stove, that might be dangerous for the child or that distract the parent’s attention away from the child.
**Risks of Use.** Parents shared numerous concerns about what their child could see on screen devices and the impact of how they use such devices.
  ***Harmful.*** Children can learn bad words and behaviors and certain types of content can cause children to be scared or stressed. Parents expressed concern that their toddler would imitate bad behaviors (e.g., violent or aggressive behavior) viewed on a screen device. Parents felt that screen content is less beneficial at this age, compared to when children are older.
    *Device-specific concerns regarding potential harm:* Parents mentioned children are likely to have sudden unintended exposure to bad content on mobile devices either with YouTube or child tapping a screen on a mobile device, with only a little mention of this related to TV (e.g., child plays with remote). A few felt that phones were acceptable since their child does not know how to access non-parent selected content or notice the apps they do not want them to access.
  ***All-consuming.*** Parents reported that screen use can consume a child’s attention, preventing engagement with other positive activities that contribute to their development (e.g., playing with toys, engaging in social interactions). Parents believed that screen use while eating distracts a child from eating and can interfere with the valued social interactions that occur at mealtimes. Many expressed a strong belief that screens should not be on during mealtimes; some reflected that this mirrored their upbringing or family traditions. When used before sleep, some parents reported that toddlers can become dependent on screens for falling asleep or struggle to fall asleep. Excess use in general was felt to be habit-forming.
    *Device-specific concerns regarding all-consuming use:* Although a few devices were identified when parents mentioned the habit-forming outcome of screen use, parents expressing this belief were most often talking about smartphones. Parents shared that the current social norm is to use smartphones and for people to be ‘stuck’ or ‘attached’ to their phones.
  ***Poor physical outcomes.*** Some participants felt too much screen use, as well as sitting too close, can negatively affect a child’s eyes. Furthermore, the sedentary aspect of screen use can negatively impact on children’s well-being, specifically if using the screen for too long. Some parents believed that on/off viewing in short bursts prevented such poor outcomes.

### 3.2. Research Question 2: What Screen-Related Parenting Practices Do Mothers and Fathers of Toddlers Use and Do These Differ by Screen Device Type?

Parents reported using a myriad of parenting practices to manage their toddler’s screen use, including some that are device-specific. Identified themes are outlined below with representative quotes in Table 3. Similar to beliefs, findings were similar across participant groups with no identified differences in screen-related parenting practices by language (Spanish, English) or parental role (mother, father).

**Close attention to content.** Parents reported that they commonly choose the content their child sees, with some allowing only educational content, cartoons, or specific channels or apps. Some parents reported pre-watching content prior to letting their child view it. Parents reported closely monitoring the content their child sees by watching with their child, listening in, being nearby, or frequently checking in on their child. Being nearby was utilized as a way to stop exposure to ‘bad’ content that suddenly appears. Some parents also reported using controls on their screen devices to limit their child’s access (e.g., YouTube kids, Netflix kids, not allowing child to use the remote control).
  *Device-specific close attention to content:* Some parents felt smartphone use needed especially close monitoring.
**Monitoring duration.** Parents reported monitoring the amount of time their child uses screen devices, and controlling exposure time in a variety of ways. Some reported allowing very short episodes of viewing. Others allow their child to decide how long to view, since their child gets bored or stops viewing or using a screen device before the parent needs to intervene. Sometimes parents determine the length of use based on how long the parent needs the child to use a screen device, for example, for entertaining their child when the parent needs to be engaged in other activities. Other times, parents reported using the program length to determine the length of viewing. Watching the clock was not widely employed.
  *Device-specific monitoring of duration:* Parenting practices specific to smartphones included hiding the smartphone and telling others to not let their child use this type of device.
**Behavior Management.** Parents use screen devices to manage behaviors such as crying, fussing, and screaming, as a way to calm their child down. Smartphones are used when away from home or in the car to manage behaviors. Parents reported doing the same in the home, but some also reported using the TV for behavior management. Some parents reported using screens to entice or reward behavior (e.g., eating, obeying), but few use screens to discipline (e.g., take away or limit use based on behavior). Parents shared that they often try other options first to entertain their child, using screens as their backup for entertaining or calming their child. Parents also reported utilizing screen devices in a supportive way to entertain or occupy their child when they need to do other things (e.g., eating, cooking, cleaning, self-grooming, errands, doing unsafe things) both inside and away from home. For some, this gives them a period to relax, handle their mood, or rest when not feeling well.
  *Device-specific use for behavior management:* Differences in screen device use related to the context of use and the mobility of mobile devices. Mobile devices were reported to function in a babysitting-like way when away from home. Smartphones were typically the device parents referred to when talking about the behavior management benefit of screen use when a child has difficult behavior—both in the home and away from home. Parents use mobile devices to reactively manage behavior when away from home.
**Co-Use.** For some parents, co-use was reported to be part of their routine. In some families, parents engage in co-use of screens to support content monitoring or learning. Parents reported interacting with their child around the content their child sees on screen devices. This included responding to child reactions (e.g., verbalizations, pointing), repeating words, and singing along with songs.
**Situation-specific parenting practices**. Parents reported different practices related to specific situations. Many parents reported having the TV on nearby while the child plays with toys, enabling the child to switch their attention between the two. Children also play near their parents while their parent(s) watch TV. Some families use screens devices around bedtime, with most reporting TV viewing. Some report TV use as part of their bedtime routine. Some parents have a clear line/rule about screens at mealtimes.
  *Device-specific parenting practices for certain situations:* Parent reports of simultaneous play while using a screen device were limited to TV use. When screens are used when eating, TV was the typical device and often was reported as a shared family activity or it was on in the background. When screens are used before sleep, most parents reported TV use.

### 3.3. Research Question 3: What Are Perceived Contextual Contributors to Toddler Screen Use in Parents of Very Young Children?

Parents endorsed a variety of contextual factors influencing child screen use. Representative quotes are in Table 4. Some parents reported that the weather contributes to their child’s screen use, with cold weather limiting outside time, and leading to increased screen use inside. Warmer weather has the opposite effect, offering the opportunity to be outside, which parents said results in less screen use. Many reported that their weekday or weekend routines shape their child’s screen use. For example, more weekday use happens when the child’s father returns home from work, or the child has very little screen use when the family has many weekend activities away from home. In some families, extended family members contribute to their child’s screen use by encouraging use or modeling use. For some parents, this is contrary to their desires. In other families, extended family disapprove of parents’ perceived permissiveness about allowable toddler screen use. Some parents felt that their neighborhood is not safe, which contributes to increased indoor time and consequently increased child screen use. One person, who mentioned that they did not live in a safe neighborhood, considered the TV a protective device, leaving it on all night to show that people are home. Others viewed their neighborhood favorably, with opportunities to be outside or engage in activities, and therefore not impacting screen use. Others reported living in apartments without yards, which they perceived as contributing to increased indoor time and consequent screen use.

## 4. Discussion

This qualitative study, guided by the social ecological model, focused on screen-related beliefs and parenting practices, as well as contextual contributors to toddler screen use in low-income Mexican American mothers and fathers of toddlers. Our findings offer interventionists and providers insight into the day-to-day management of screens in the lives of this population. In general, parents are thoughtful about screen use in this age group and report using a multitude of parenting practices to manage use and minimize perceived risks, with some differences based on screen device type. The findings on parental perceptions of contextual contributors also enhance our understanding of the factors parents consider influential in their child’s screen use. Given that about 16% of children in the US are of Mexican descent, half of whom live in low-income households, these findings are relevant to a large and growing population [34,35].

Addressing our first research question regarding parental beliefs, parents perceived numerous benefits and risks to their toddlers using screen devices. Parents believe their children are learning while using screen devices. This is a belief that exists across demographic groups, including different child age groups, racial and ethnic groups, and family SES levels [7,8,9,10,14,36]. We found that parents believe that screens can be helpful for parents. This is also widely endorsed in other studies, especially in parents of young children [9,14,36,37,38]. Parents also have many concerns about the harmful effects of screen use, including both mental and physical effects. Again, such concerns are broadly shared by parents [8,9,10,14,36]. The similarity of our findings to those of other studies suggests shared parental beliefs about screen use across demographic groups. That said, the developmental stage of toddlers appeared to influence some beliefs. For example, a few parents expressed that they were not concerned about screen use since they did not feel that their child could understand or remember the content viewed. Additional work is needed to evaluate how the beliefs reported by parents in this study are associated with actual toddler screen use, in order to inform intervention design.

Findings related to our second research question on parenting practices suggest that parents manage these perceived risks and benefits using various parenting practices. Given the high levels of concerns regarding content, it is not surprising that parents focus on controlling the content their child sees. Parents managed content through close monitoring, with some reporting use of specific controls (e.g., YouTube kids, Netflix kids, blocking sites). Parents also monitored the duration of screen use, which for some involved relying on their toddler’s eventual boredom with content and desire to do a different activity. Monitoring of content and duration are common parenting practices [8,10,32,39,40]. Parents used screen devices as tools for managing child behavior; this is also commonly endorsed in other studies, especially in parents of young children [9,14,41,42]. The similarity of our findings with those of other studies suggests that parents across demographic groups have similar experiences managing their child’s screen use. However, parents in this study did not endorse using screens for discipline or punishment, although parents of older children have reported this practice [9]. The relationship between the above parenting practices and toddler screen use warrants further investigation given the dearth in the literature on this topic. Such research could help medical providers and interventionists determine where to focus their efforts to promote healthy screen use in this age group.

Embedded in our first and second research questions was the question of whether parental beliefs and parenting practices vary by device type. This study’s exploration of parental beliefs and practices across screen device types offers nuanced insight into how parents of toddlers view and manage different device types within their own family. As previously stated, most research on screen use has focused on only one screen device type (e.g., TV or mobile devices) or on screens as a general category [9,36,43]. Parents in this study reported a high level of specific concern regarding smartphones and their potential impact on their toddler, but they also endorsed the value of their child learning to use this technology. Parents also appeared to manage smartphones differently than TV. Parents reported close monitoring of smartphone content due to easy access to or sudden exposure to inappropriate content. Despite this concern and the close monitoring, smartphones were the screen device parents used for babysitting and behavior management. A 2016 qualitative study focused on mobile device use in early childhood reported similar findings in a diverse sample of 35 parents [36]. In conversations about certain daily activities—eating, sleep, co-use, and playtime—parents in this study focused their discussion on TV use. Together, these findings suggest the need to tailor counseling on healthy screen use by device type and to recognize that specific situations may be associated with certain types of screen use.

Further understanding the ecology of screen use, as asked in our third research question, parents offered insight into contextual factors that may contribute to toddler screen use in their homes. Family routines, weather, and extended family may play a role in toddler screen use, as well as neighborhood factors and the built environment. A study by Lindsay et al. reported similar findings on the influence of extended family and weather on screen use from interviews with Brazilian immigrant families with preschoolers [14]. Neighborhood factors influence individual behavior, so it is not surprising that parents identified this as a factor [44]. For many Mexican American families, living in disadvantaged neighborhoods is a reality, and parenting must be adapted to this context [45,46,47]. Future research is needed to evaluate the impact of neighborhood factors on screen use in this age group.

The inclusion of fathers in this research offers important contributions to the literature. Very little is known about how fathers shape child screen use, yet early qualitative and quantitative results suggest they play an important role [9,48,49]. For example, Jago et al. reported that paternal TV viewing time was associated with child TV viewing in children 3–10 years old [48]. Findings from our study suggest similarities across maternal and paternal screen-related beliefs and parenting practices. The widespread use of screen devices possibly drives the shared beliefs and practices across these parental roles. Ochoa et al. reported in a small sample (*n* = 40) of Latine parents of mixed SES with children less than 5 years old, that fathers, more often than mothers, reported the importance of continuous monitoring to prevent exposure to inappropriate content on mobile devices [10]. We did not find a difference between mothers and fathers on this topic, but rather that both mothers and fathers similarly felt that close monitoring was needed. Demographic differences between our study participants and those of Ochoa’s study (e.g., SES, focal child age, Latino subgroup) could contribute to these divergent findings. Further research should evaluate whether mothers and fathers quantitatively differ in their endorsement of specific beliefs or their use of certain parenting practices, in addition to evaluating the relationships of such beliefs and parenting practices with child screen use. In addition, the relative influence of mothers and fathers on specific types of screen use should be examined. Future research could also assess the degree to which mothers and fathers agree about their toddlers’ screen use, as a possible predictor of actual screen use.

Providers and interventionists aiming to support healthy screen use in this population should consider the beliefs, practices, and perceived contextual influences identified in this study. Parents expressed a high level of concern about screen content and they employed a variety of parenting practices to manage screen use. When counseling, providers can promote healthy screen use by reinforcing parental desires for the child to learn, yet not be harmed by screen devices, while also recognizing contextual factors that may influence use. Providers could praise families, when applicable, for their close monitoring of content viewed, possibly reminding families that content viewed on any screen device should be of high-quality. Limiting screen use for behavior management is also important. Providers may want to focus on helping families to develop behavior management strategies that do not involve screen use, especially for situations when the smartphone is typically used. That said, the use of screen devices to occupy a child while the parent gets something done should be recognized as, at times, the best and possibly only approach a parent may have to manage the multiple demands they juggle. Counseling families to anticipate tantrums in reaction to limit setting (e.g., limiting duration of use or content used) may also be helpful, as these are commonly reported reactions [25]. Finally, conversations around screen use in specific situations, may need to focus on specific device types, such as turning off the TV at mealtime even when it is not on for the child.

This qualitative study has several limitations. The research focused on Mexican Americans living in the greater Denver metropolitan area. These findings may not apply to families in other populations or geographic regions. Furthermore, participants were recruited in a federally qualified health center serving low-income families. These findings therefore may not generalize across socioeconomic strata. Finally, evidence suggests that screen use has increased in families since the start of the COVID pandemic [50]. Whether and how parental beliefs and practices regarding screen use may have changed since then is currently unknown. Many contextual factors have been in a state of change since the start of the pandemic, with ongoing change dependent on future reactions to the pandemic.

## 5. Conclusions

Given the evidence that screen use in children and adults has increased since the start of the COVID pandemic, more attention should be given to supporting families to engage in healthy screen use, especially those families at higher risk of poor screen-related outcomes. Providers can build on parents’ goals and experiential realities to support parental self-efficacy in managing child screen use as well as children’s behavioral challenges. The social ecological model provides a framework for understanding the multiple levels of influence on toddler screen use behaviors, including parental beliefs and parenting practices as well as contextual contributors as outlined in this study. This study’s findings extend the current literature to a population at risk for unhealthy screen use. Our device-specific findings suggest that interventions promoting healthy screen use in this age group may need to consider the possibility that beliefs and management of screen use may vary by device type. Additional work is needed to examine, in both mothers and fathers, the relationship of the identified screen-related beliefs and parenting practices with actual toddler screen use. Future research should also examine the influence of contextual factors on parenting and early screen use. In this era of pervasive screen use, such research will provide critical information for the development of both culturally and contextually tailored interventions to promote healthy screen use in this population.

**Note:** The terms “Latino” and “Hispanic” are widely used in health research in the United States (US), often interchangeably, and refer to individuals of Latin American and/or Iberian descent residing in the US [51,52]. The Latino/Hispanic population is heterogeneously characterized by variations in race, heritage, and linguistic preferences [53]. In this paper, we have used the original terminology of the research we have cited. Those authors may refer to their samples as Latino, Latinx, Latine, Hispanic, or Mexican American. Latinx and Latine are recent gender-neutral terms for Latino. In the current study, we use the term “Mexican American” to refer to participants who reported heritage from Mexico. We have used the term “Non-Hispanic White” to refer to individuals who identify their race as White and who do not identify as Latino or Hispanic [54]. Race and ethnicity, including the terms Latino, Latinx, Latine, Hispanic, Mexican American, and White, are self-reported identities in the US).

## Figures and Tables

**Table 1:** Participant Demographics and Interview Characteristics for In-depth Interviews with Mexican American Parents of Toddlers (*n* = 32 Participants) *.

Participant Characteristics	Percent (*n*) or Mean (SD)
Mean age (years)	29.22 (6.4)
Education level	
≤High school degree	75% (*n* = 24)
Employed	69% (*n* = 23)
Household characteristics **	Percent (*n*) or mean (SD)
Partnered	75% (*n* = 21)
Mean age of focal child (months)	19.7 (3.7)
Mean daily hours of focal child screen use, weekday	2.1 (1.4)
- Range (hours)	0.17 to 6 h
Mean daily hours of focal child screen use, weekend	1.7 (1.4)
- Range (hours)	0 (*n* = 2) to 5 h

SD = Standard deviation. * Table adapted from reference [25]. ** Four fathers were partnered with participating women (two women interviewed in English and two interviewed in Spanish), thus participants represented twenty-eight households.

**Table 2:** Screen-related Beliefs: Representative Quotes from Interviews with Mexican American Parents of Toddlers (*n* = 32) *.

General and Device-Specific Beliefs	Quotes
Benefits of use	
Learning	So, he’s learning to talk more when he’s on the phone because he tries to repeat things. Or sometimes he’s talking to Siri, and he’s trying to make a conversation up with her. And so he tries to talk more. Because when we tell him [to talk], he just starts laughing, or he runs away, and we’re like, “Come back. We have to talk more.” And with the phone, he does that.
	Device-specific quote: Well, I like it (the smartphone) because she learns about technology, she starts to move it around, like “Oh look, and she slides this one.” “Oh, look, this one moved here.”
Enjoyment	We are with one each other, and he’s like Mommy this, Mommy that, or like telling me about what’s happening in his own way. So, he’s interacting with me about things he’s watching. So that’s something that I like about him because we’re making a bond. And there are times that she’ll climb up with her dad and they’re watching TV together and when someone falls or comes out of water, she gets excited and starts to imitate what my husband does. And so, I say that it’s a benefit, that it’s moment of father and daughter.
	Device-specific quote: It’s just ‘us time’. We get to bond. We get to just have us time. (referring to co-use of TV between participant and child)
Helpfulness	That he is not screaming at the top of his lungs and lets me do what I have to do. I guess it [smartphone use] is good for both of us. He’s not screaming, throwing tantrums, throwing himself. And I’m able to complete what I have to do. I guess too, like quiet time when he’s extremely exhausted and needs a little bit of quiet time and space. Keeping him entertained lets everybody kind of chill. Instead of Archie running around, crying to be in arms or screaming at everybody, or throwing—he does throw toys at people. So, he’s not throwing toys at people and biting and being mean. Sometimes he just needs that chill.
Risks of Use	
Harmful	Sometimes children imitate what they watch. Sometimes, they could get a knife, or they could grab anything, and that’s what concerns me.
	Device-specific quote: Even if you choose kids videos for them [on the phone], eventually videos will come out that are not appropriate for kids.
All-consuming	I think definitely less than an hour. Just so they don’t become dependent on it. Because they’re so little, and their brains are developing. There is so much other stuff for them to do. He’ll stay up watching it and watching it, and I don’t know, but yeah, like he sometimes struggles more to fall asleep or I struggle more to get him to fall asleep when he has just finished watching TV. I feel like they don’t eat properly when they’re watching the TV. They get distracted with it. So I feel like they won’t eat all the things they need, or like how they’re supposed to, for, like, because they’re watching TV. Or they’re like, “I’m full”, because they want to watch the TV. There are some kids, I think, that just don’t want to engage in anything. If Victoria was watching too much TV, I think she wouldn’t engage in anything.
	Device-specific quote: I feel like they get an addiction. I feel like I have an addiction to my phone. Not like an actual addiction, but I feel like… they’ll get used to it more and expect it more.
Poor physical outcomes	So, it’s not a long time so I feel like it’s not going to damage her eyes or anything.

* All names are pseudonyms.

**Table 3:** Screen-related Parenting Practices: Representative Quotes from Interviews with Mexican American Parents of Toddlers (*n* = 32) *.

General And Device-Specific Parenting Practices	Quotes
Close attention to content	I make sure it’s cartoons, something she’ll focus her attention on and something that’s appropriate for her age. I took the videos that she does watch now, I have gone through every single one of them to make sure that they were safe, didn’t have like spam. I’ll never leave her with the phone for more than 10, 15 min by herself. I’ll—“let me see, let me see, let me make sure you have what you need to have on there, because if not, you’re not watching it anymore”. Device-specific quote: That’s what I don’t like about devices, even if you block it, pages keep coming up, I have no idea why this happens. (Referring to smartphone)
Monitoring duration	Sometimes I’ll just like whenever I feel like she’s been on the phone for too long, I tell her that I’m just going to take it away. I guess by the time it took me to clean. By that time like, “Oh, okay. It takes me 25 min to clean, that’s how much time she’s gonna spend on the tablet.” Time wise—I feel like she just kinda cuts herself off. She doesn’t really—I feel like I’m not really on my phone that much, so she doesn’t even really care to look at the screen that long. Once she—“Oh, okay. That’s enough.” She’ll just drop it. Device-specific quote: That sometimes she wants to have it for more time than what I allow her. And she throws a tantrum, that’s why I take [the smartphone] from her, I barely let her use it and hide it.
Behavior Management	I’m able to clean and cook and get chores and everything handled that I need to, you know. And I don’t have to worry about her moving. Or her getting into anything. If I put her on her videos, I’m able to at least get some stuff done that I need to get done. So, I don’t have to be like “Don’t do that! Don’t get into that! Stay still just for one second.” Because it’s mostly when we are in the car that he gets like a little cranky, and he wants to get up out of the car seat. So, it helps me in that way, that I have to be driving, and I just hand him the phone so he can stay a little bit calm, and he doesn’t be like crying a lot or trying to get out of the car seat. Device-specific quote: Just when she’s really fussy and we’re out and about. That’s when I give her my phone.
Co-Use	I would prefer her to use it with me, not right next to me, but just while I’m there, so if anything I’m able to like see what she’s exposed to. When he is with me, I try to watch entertaining stuff, funny things or music, so that he has fun too. Device-specific quote: It’s just ‘us time’. We get to bond. We get to just have us time. (referring to co-use of TV between participant and child)
Situation-specific Parenting Practices	Some days, she’ll watch an episode, so maybe 20, 30 min. She’ll be in and out of it, though. She’ll never sit there and watch the entire thing. She’s busy wrapping her babies and then looking at the babies and then waiting for a scene of the babies to come up. I’ll turn it on to cartoons like Moana. And he’ll sit for a couple minutes, go play, come back, sit. So, he’s not just like sitting in front of the TV. Sometimes, when he’s not tired, like if he had a long nap in the evening, I will let him unwind with Moana. He’ll lay on my bed. But it’s not so much that he’s playing. He’s just laying there watching it. But then once I notice that he’s getting tired, then I just turn it off and put him in his crib because I don’t want him to get used to having to watch that to go to sleep. I never allow them to do that. I just let them eat first or take the meal or whatever they’re doing, and then come and watch TV. Device-specific quote: He keeps playing, sometimes the TV is on all day, so he sometimes goes back and forth, he remembers that the TV is on and returns to watch it, then, he continues playing his games, and then, later, if he hears a song he likes and returns to watch TV or videos again; and that’s how it is all day. Like when we get out of work and the food’s already ready, sometimes we will turn [the TV] on and watch it all of us together. But we try not to do that because he won’t eat. But sometimes if he can’t sleep, I let him have it. I’m very tired, and just to keep him in the room, I turn on the TV for a while. And after that, I say: “Okay, turn it off, its bedtime!”. And he stays put.

* All names are pseudonyms.

**Table 4:** Contextual contributors to screen use reported in In-depth interviews with Mexican American parents of toddlers (*n* = 32 Participants).

Contextual Contributors	Representative Quotes
Weather	And the advantages would be that it’s something we can use to entertain them when, for example, the weather doesn’t allow us to go outside.
Weekday or weekend routines	During the weekend we go out, we barely watch television or—the phone, I don’t really like to let her use it.
Extended family members	But we’ve tried to be more outspoken about it with people we know, and we let them know we don’t really agree with having her use a smartphone, because then we struggle with tantrums because she sees that when—if they let her use it before, then she wants to take it from us too and throws a tantrum because of it.
Neighborhood	No, I think it’s beneficial because there are a lot of kids, and when she hears the kids outside, she wants to go outside. So, she doesn’t pay any attention to the TV or the phone, she wants to go outside. So I would say it’s a benefit where I live. I think that that affects her community; I mean her screen time because instead of her being out and playing she’s inside because I don’t want to be outside because I don’t like my neighborhood.
Housing	In order for me to take them out to play I have to go to the park. There is no garden where he can go out and he’s like… it’s not the type of house where you can walk around doing that… so, maybe it influences that he’s more shut in, due to the fact that it’s an apartment.

## Data Availability

The data presented in this study are available on request from the corresponding author once a data-sharing agreement is in place. The data are not publicly available due to the possibility of deductive disclosure of subjects.
